# NEUROPSYCHIATRIC SYMPTOMS IN PRIMARY CARE PATIENTS WITH DEMENTIA

**DOI:** 10.1093/geroni/igac059.2378

**Published:** 2022-12-20

**Authors:** Constanca Paul, Laetitia Teixeira, Susana Sousa

**Affiliations:** University of Porto, Porto, Porto, Portugal; University of Porto, Porto, Porto, Portugal; ICBAS, University of Porto, Porto, Porto, Portugal

## Abstract

Neuropsychiatric Symptoms of dementia are mostly valued within care and burden of formal and informal caregivers and less considered as diagnosis predictors. Diagnosis of dementia in Primary Care Service (PCS) is frequently late and based mostly in general clinic assessment, and patient/family subjective complaints. We study the association of Neuropsychiatric Symptoms (NPI-Q) and existing diagnosis of dementia in PCS. The objectives are to know 1) the prevalence of symptoms identified by familial caregivers in people with a diagnosis of dementia and 2) if there is an association of symptoms with the existence of a diagnosis.Method: we randomly select a community based sample of a pool (N=2734) of primary care users with mental health concerns referred by General Physicians, (N=154), mean age 76 years (sd 7.8), 57% women. Caregivers (N=39) were interviewed and fulfil NPI-Q. Results of descriptive and logistic regression analysis showed that 39% (60) had a formal diagnosis of dementia not differentiating men and women. The neuropsychological symptoms frequency varied between 3.1% (hallucinations) and 16.3% (apathy/indifference), and the symptoms’ mean was 4.5(sd 2.1). The amount of symptoms was not associated with the diagnosis. The symptoms that predict the diagnosis were Apathy/ Indifference OR 5.24(1.25-22.0),p.024 and Motor Disturbance OR 5.70(1.17-27.6), p.031. Qualitative data from caregivers interview show that they are not very comfortable with the terminology of NPI-Q, which may limit the accuracy of assessment.

Conclusion some neuropsychological symptoms identified by caregivers seems to be relevant as predictors of diagnosis of dementia in Primary Care.

